# Ultrasound Neuromodulation as a New Brain Therapy

**DOI:** 10.1002/advs.202205634

**Published:** 2023-03-24

**Authors:** Roland Beisteiner, Mark Hallett, Andres M. Lozano

**Affiliations:** ^1^ Department of Neurology Functional Brain Diagnostics and Therapy High Field MR Center Medical University of Vienna Spitalgasse 23 Vienna 1090 Austria; ^2^ Human Motor Control Section National Institute of Neurological Disorders and Stroke National Institutes of Health 10 Center Drive Bethesda MD 20892–1428 USA; ^3^ Division of Neurosurgery Department of Surgery University of Toronto Toronto ON M5T 2S8 Canada

**Keywords:** brain stimulation, brain therapy, focused ultrasound, transcranial pulse stimulation

## Abstract

Within the last decade, ultrasound has been “rediscovered” as a technique for brain therapies. Modern technologies allow focusing ultrasound through the human skull for highly focal tissue ablation, clinical neuromodulatory brain stimulation, and targeted focal blood‐brain‐barrier opening. This article gives an overview on the state‐of‐the‐art of the most recent application: ultrasound neuromodulation as a new brain therapy. Although research centers have existed for decades, the first treatment centers were not established until 2020, and clinical applications are spreading rapidly.

## Graphical abstract

Ultrasound has been “rediscovered” as a technique for brain therapies within the last decade. This article gives an overview on the state‐of‐the‐art of the most recent application: ultrasound neuromodulation as a new brain therapy. Although research centers have existed for decades, the first treatment centers were not established until 2020, and clinical applications are spreading rapidly.

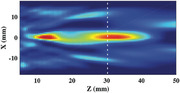

## 1 Introduction

Within the last decade, ultrasound has been “rediscovered” as a technique for brain therapies. Notably, the very first medical applications of ultrasound were neurological applications: Pohlmann et al.^[^ ^1^ ^]^ tried to treat patients with neuralgia, and Dussik et al.^[^ ^2^ ^]^ generated the first ultrasound image, showing the lateral ventricles of the human brain. Modern technologies allow focusing ultrasound through the human skull and enable non‐invasive stimulation or ablation of brain tissue. Clinical neuroscientific studies have shown that: a) highly focal tissue ablation (e.g., tremor therapy); b) clinical neuromodulatory brain stimulation (e.g., Alzheimer's therapy); and c) targeted focal blood‐brain‐barrier opening (e.g., focal drug transfer) are possible. Meanwhile, ultrasound surgery and, just recently, ultrasound neuromodulation have entered routine clinical therapy.^[^ ^3^, ^4^ ^]^ The rapidly ongoing methodological and clinical progress opens completely novel perspectives for ultrasound brain therapy. This is important since brain diseases are one of the most urgent problems in our rapidly ageing society. Effective medications are often missing (e.g., for dementias) and application of surgery is limited due to invasiveness, particularly in the elderly. In light of increasing therapeutic application of ultrasound neuromodulation and therapeutic patient requests, this article focuses on ultrasound neuromodulation as a new brain therapy. Although research centers have existed for decades, the first treatment centers were established in 2020 and currently no detailed overview over clinically applicable technologies, current therapeutic results, and therapeutic benefits compared to electromagnetic therapies exist.

## 2 Methodology of Clinical Ultrasound Neuromodulation

Three technical approaches have been used to modulate human brain activity. The first approach comprises standard diagnostic systems built for Transcranial Doppler Sonography to monitor and diagnose the intracerebral blood flow situation. Although they have a limited field of view, they cannot be focused to a small brain area. The second approach is highly focused systems, which can target stimulation to very small brain areas. The third approach is highly focused and individually navigated systems, which allow us to precisely target individual brain areas on individual magenetic resonance (MR) images. Since every brain is different and pathologies may result in gross morphological brain changes, precise targeting capabilities are essential. Therefore, highly focused navigated systems are clearly state‐of‐the‐art for clinical ultrasound neuromodulation.

**Figure 1:**
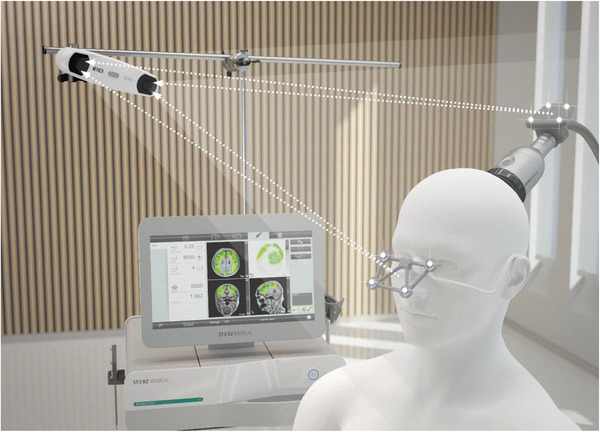
Treatment setting for one of the current state‐of‐the‐art navigated and highly focused neuromodulation systems (TPS system). Reproduced with permission. Copyright 2022, Storz Medical AG.

For highly focused ultrasound systems two different classes exist. The first class builds on diagnostic ultrasound and uses sinus tones (single ultrasound frequencies) in the range of several hundred kilohertz. This approach is described as Focused Ultrasound (FUS). Typically, FUS sinus tones are presented in a pulsed mode, which means short trains of ultrasound (e.g., 100 ms) are followed by silence (e.g., 300 ms). The relationship between the ultrasound train and the following pause before the next ultrasound train defines the duty cycle (here 25%). In the literature an extraordinary variability in sonication schemes has been described, including a blocked presentation of duty cycles which may then be paused for a longer time (seconds) and thus generate longer second order stimulation pulses (e.g.,^[^ ^5^ ^]^). For neuromodulatory effects, such sonication patterns are used over several minutes. Meanwhile, various FUS systems are in human and clinical use and are also produced by the medical industry. The second class of highly focused systems is a quite new neuromodulation approach. It has first been published in 2019 after a development period of about 10 years (**Figure** **1**).^[^ ^6^ ^]^ The principle builds on shock wave technologies and consists of ultrasound pulses consisting of various frequencies (frequency mixtures instead of a sinus tone). Compared to FUS, the special feature of these pulses is that they are ultrashort pressure pulses (around 3 µs) which generate stronger mechanical irritation at the highly focal brain target. The approach has been named Transcranial Pulse Stimulation (TPS).^[^ ^6^ ^]^ TPS pressure pulses are typically repeated at frequencies between 1 and 8 Hz. Besides application frequency, pulse energy (up to 0.25 mJ mm^−2^ energy flux density) can be varied. Comparable to FUS, TPS is applied for several minutes to achieve neuromodulatory effects. Currently, one TPS system is produced by the medical industry, and this is approved for Alzheimer's therapy (Conformitè Européene (CE) certification) and clinical research (Food and Drug Administration (FDA) Investigational Device Exemption (IDE) certification).

**Figure 2:**
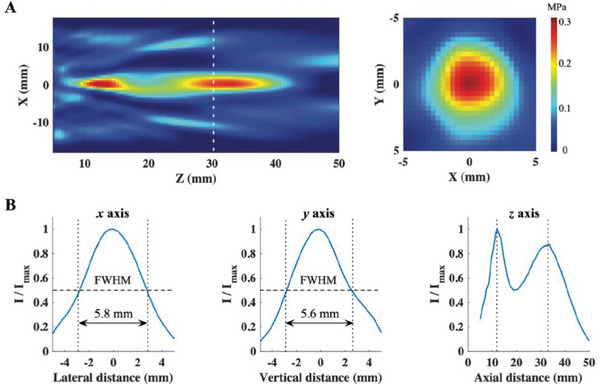
Example for a very small ultrasound neuromodulation focus. A,B) Reproduced with permission.^[^ ^9^ ^]^ Copyright 2021, American Neurological Association. Ultrasound pressure field. A) Acoustic intensity profile of the 0.5 MHz transducer with focus 30 mm measured in free water. The white line in the longitudinal maps (left) indicates the focal plane where the spatial peak pulse average intensity of the acoustic field was measured. Acoustic beam cross‐section of the focal plane is illustrated at right. B) Line plots illustrate the lateral (*x*, left) and vertical (*y*, middle) peak normalized acoustic intensity profiles for the acoustic beam in the focal plane. The lateral and vertical dimensions of acoustic beam cross‐sections measured at the intensity full width at half maximum (FWHM) were 5.8 and 5.6 mm. The line plot for the axial (*z*, right) peak normalized intensity profiles shows a near‐field peak at 12 mm and a far‐field peak at 33 mm (close to the focal length of 30 mm).

Both approaches for highly focused ultrasound neuromodulation generate very small stimulation foci. Although size of stimulation foci depend on transducer design and frequency, typical foci are cigar shaped with a length of around 3–5 cm and a width of about 4 mm full width at half maximum (FWHM) (examples in **Figure** **2** and Beisteiner et al.^[^ ^6^ ^]^). The TPS system and some FUS systems represent highly focused and navigated state‐of‐the‐art systems. Their ultrasound focus can be navigated to cortex and deep brain areas—in real‐time and based on individual brain MR images. This allows precise modulation of small‐scale neuronal networks, including the option to uncover new circuits.^[^ ^7^, ^8^ ^]^ The features also enable personalized precision medicine.

Concerning possible mechanisms for ultrasound mediated neuronal activation changes, complete knowledge is still lacking. Recent data indicate direct mechanically induced depolarization effects.^[^ ^10^ ^]^ Ultrasound can produce very small cell membrane deflections (150 nm) which change membrane voltage and lead to subsequent depolarizations. Other work on disconnected single neurons implies that ultrasound effects are stronger with higher peak pressures and effects are also evident with ultrashort pressure pulses (4 µs) as applied by TPS.^[^ ^11^ ^]^ Previously published hypotheses on mechanisms for neuromodulation include mechanical and thermal influence on ion channels of the cell membrane and effects on mechanosensitive receptors (for review see Refs. [12, 13]) Transcranial ultrasound applied to the human motor cortex depresses excitability during a single pulse of stimulation, but without after effect or effect on the contralateral motor cortex.^[^ ^14^ ^]^ Even repetitive single pulses had no aftereffect; however, when pulses were given in theta burst format, there was an increase in cortical excitability that lasted up to 30 min.^[^ ^9^ ^]^ Hence, it does seem possible to induce plasticity in the brain with transcranial ultrasound.

## 3 Ultrasound Neuromodulation – Differences Compared to Electromagnetic Stimulation

Clinical neuromodulation has already been performed for decades with electromagnetic techniques (see Bhattacharya et al.^[^ ^15^ ^]^ for review). Most important technologies are Transcranial Magnetic Stimulation (TMS), Transcranial Direct Current Stimulation (tDCS) and, more recently, Transcranial Alternating Current Stimulation (tACS). Therefore, the question arises which clinical benefits may be expected from ultrasound. The answer is threefold. First, the narrow focus of ultrasound neuromodulation provides an unprecedented precision for targeting small brain areas. Second, ultrasound is the first technique, which allows for non‐invasive, selective and focal deep brain stimulation.^[^ ^16^, ^17^ ^]^ Currently, focal deep brain stimulation is only possible via implantation of deep electrodes. Noninvasive TMS and tDCS must always affect the cortical surface. With the use of temporally interfering electric fields, it might be possible to target depth, but this is still experimental, involves exposure of large non‐targeted brain areas to high frequency fields and all details are not fully resolved.^[^ ^18^ ^]^ Third, clinical neuromodulation requires stimulation of pathological brains. This includes major changes of the normal conductivity situation inside the brain. For electromagnetic techniques, it is nearly impossible to always correctly model this changed conductivity for precise individual targeting.^[^ ^19^ ^]^ In contrast, ultrasound targeting is not affected by conductivity changes.

Very recent developments now indicate that strengths of ultrasound and electromagnetic techniques may be combined. A promising new development with electromagnetic brain stimulation is closed loop neuromodulation. For this, electromagnetic brain activity is recorded, and stimulation parameters are then adapted to the recorded signal.^[^ ^20^ ^]^ Brain activity and brain rhythms may thus be modulated via feedback loops. New animal data indicate that closed loop neuromodulation might also be possible using electromagnetic signals as input and highly focused ultrasound systems as stimulation output.^[^ ^21^, ^22^ ^]^

## 4 Clinical Effects with Ultrasound Neuromodulation

The first clinical study with a focused ultrasound system (non‐navigated TPS precursor) was performed by Lohse‐Busch et al.^[^ ^23^ ^]^ in patients with disorders of consciousness (apallic syndrome). Shortly before, another patient study—however using a non‐focused diagnostic system – was published.^[^ ^24^ ^]^ Overall, 16 patient studies have been published, applying systems from 10 different laboratories. Fourteen of these 16 studies report significant clinical effects, with 13 describing (at least partial) patient benefits (see **Table** **1**). In addition, a very recent analysis of uncontrolled TPS data from a treatment series in parkinsonian (PD) patients found strong improvements in PD clinical scales.^[^ ^25^ ^]^ In this work, 2 weeks of TPS treatment improved Unified Parkinson's Disease Rating Scale (UPDRS) I–IV total scores from a group mean of 30.4 to 23.8 (*p* < 0.0005, paired *t*‐test, 2‐sided). It is important to note that some of the studies include independent neurophysiological support for the clinical ultrasound neuromodulation effects. Using the MR technique of asymmetric spin echo labeling, Nicodemus et al.^[^ ^26^ ^]^ report a local perfusion increase after sonication with a non‐focal diagnostic system. Beisteiner et al.,^[^ ^6^ ^]^ Popescu et al.,^[^ ^27^ ^]^ Matt et al.^[^ ^28^ ^]^ and Dörl et al.^[^ ^29^ ^]^ applied task‐based functional magnetic resonance imaging (fMRI), resting‐state fMRI and MRI cortical thickness analyses. They report brain activation increases in task specific brain areas, connectivity increases within the diseased brain network, and reduced brain atrophy in disease specific brain areas. Jeong et al.^[^ ^30^, ^31^ ^]^ used ^18^F‐fluoro‐2‐deoxyglucose positron emission tomography (PET) to monitor the regional cerebral metabolic rate of glucose (rCMRglu). They found significant regional cerebral metabolic rate of glucose (rCMRglu) increases after sonication. Lee et al.^[^ ^32^ ^]^ recorded electroencephalographic data from deep and superficial electrodes and described changes of neuronal activity during and after sonication. Wang et al.^[^ ^33^ ^]^ report stronger electroencephalography (EEG) P300 latency reduction and amplitude increase after ultrasound neuromodulation. Cain et al.^[^ ^34^ ^]^ report fMRI signal decreases of the sonicated thalamus during sonication and a correlation between clinical recovery and fMRI connectivity changes.

**Table 1:** Publications on clinical ultrasound neuromodulation. Clinical results based on state‐of‐the‐art focused and navigated stimulation are highlighted in bold

Studies	Systems	Targets	Specifics	Clinical effects
1) Hameroff et al.^[^ ^24^ ^]^	Diagnostic system (GE‐LOGIQe)	Inferolateral frontal lobe	31 patients with chronic pain	Pain reduction in back pain patients, affect improvement
2) Lohse‐Busch et al.^[^ ^23^ ^]^	Focused transcranial pulse system (TPS Precursor, Storz Medical)	Global stimulation	5 patients with apallic syndrome	Coma scores improved in unresponsive wakefulness patients
3) Monti et al.^[^ ^35^ ^]^	Focused navigated system (Brainsonix, MRI compatible)	Thalamus	1 patient with post‐traumatic disorder of consciousness	Recovery from brain injury
4) Nicodemus et al.^[^ ^26^ ^]^	Diagnostic system (DWL Doppler Box X)	Mesial temporal lobe (Alzheimer's disease), substantia nigra (Parkinson's disease)	11 patients with Alzheimer‘s disease, 11 patients with Parkinson‘s disease	Majority of patients showed improved clinical sores. Clinical results supported by ASL MRI Perfusion increase in 2/22 patients
**5) Beisteiner et al**.^**[**^ ^**6**^ ^**]**^	**Focused navigated transcranial pulse system (TPS, Storz Medical AG)**	**Dorsolateral prefrontal cortex, default mode network**	**35 patients with Alzheimer‘s disease (multicentric)**	**Improved cognition scores (CERAD) over 3 months/atrophy reduction (Popescu et al**.^**[**^ ^**27**^ ^**]**^ **)/Depression reduction (Matt et al**.^**[**^ ^**28**^ ^**]**^ **). Neuropsychological results supported by morphological, task based and resting state functional MRI**
6) Brinker et al.^[^ ^36^ ^]^	Custom focused navigated system	Hippocampus	1 patient with drug resistant epilepsy	Not analyzed
7) Reznik et al.^[^ ^37^ ^]^	Focused system (Neurotrek U+, Neurotrek Inc.)	F8 (right frontal EEG Position)	24 students with depression, sham controlled	No depression change, trait worry decreased
**8) Jeong et al**.^**[**^ 30, 31 ^**]**^	**Focused navigated system (NS‐US100, Neurosona Co., Ltd.), microbubble contrast agent used**	**Right hippocampus**	**8 patients with Alzheimer‘s disease**	**Improved verbal learning tests. Neuropsychological results supported by rCMRglu PET**
**9) Stern et al**.^**[**^ ^**38**^ ^**]**^	**Focused navigated system (BX Pulsar, Brainsonix, MRI compatible)**	**Anterior temporal lobe (tissue resected after treatment)**	**8 patients with drug resistant epilepsy**	**Slight performance decrease in 1 test. Histological analysis of sonicated tissue without ultrasound damage**.
**10) Cain et al**.^**[**^ ^**39**^ ^**]**^	**Focused navigated system (Brainsonix, MRI compatible)**	**Left thalamus**	**3 patients with disorders of consciousness**	**2 patients showed improved responsiveness**
**11) Lee et al**.^**[**^ ^**32**^ ^**]**^	**Focused navigated multichannel system (NaviFUS corporation)**	**Individual seizure onset zone**	**6 patients with drug resistant epilepsy**	**Seizures decreased in 2 patients and increased in 1 patient. FUS effects monitored via EEG (including depth electrodes)**
**12) Cont et al**.^**[**^ ^**40**^ ^**]**^	**Focused navigated transcranial pulse system (TPS, Storz Medical AG)**	**Dorsolateral prefrontal cortex default mode network, bilateral temporal**	**11 patients with Alzheimer‘s disease**	**Improved cognition scores (ADAS, ADAS‐Cog) immediately post treatment**
13) Wang et al.^[^ ^33^ ^]^	Focused system (Shengxiang Technology)	5 probes on forehead	30 patients with post‐stroke cognitive impairment: verum group (20 min stimulation), sham group (30 sec stim.)	Improvements in: cognition scores (mini‐mental state examination, Montreal‐Cognitive‐Assessment), Barthel Score, EEG P300 values, BDNF levels
**14) Cain et al**.^**[**^ ^**34**^ ^**]**^	**Focused navigated system (Brainsonix, MRI compatible)**	**Central thalamus**	**11 patients with disorders of consciousness**	**Significant group improvements in coma recovery scale revised. Correlation between recovery and fMRI connectivity changes**
**15) Shimokawa et al**.^**[**^ ^**41**^ ^**]**^	**Custom diffusion type system**	**Whole brain**	**10 patients with Alzheimer‘s disease, sham control (5 patients)**	**Tendency for better ADAS cog outcome with verum stimulation**
**16) Cheung et al**.^**[**^ ^**42**^ ^**]**^	**Focused navigated transcranial pulse system (TPS, Storz Medical AG)**	**Left dorsolateral prefrontal cortex (DLPFC)**	**30 patients with major depressive disorder, controlled via waiting list group**	**Significant improvements on Hamilton depression rating scale‐17 (HDRS‐17)**

## 5 Safety of Clinical Ultrasound Neuromodulation

Depending on the amount of energy transferred to the brain tissue, ultrasound applications may produce local heating and local cavitations (expansion and then collapse of local tissue exposed to a tensile pressure). The consequences may be cell damage and local bleeding. When ultrasound contrast agents are used or local gas bodies exist, the risk for cavitations is considerably increased.^[^ ^43^ ^]^ Therefore, systems used for clinical ultrasound neuromodulation have been limited in energy output. For diagnostic ultrasound, guidelines for maximal exposure limits for medical ultrasonic devices have been published by the Food and Drug Administration (FDA, USA). However, they are not applicable for neurostimulation purposes. To achieve ultrasound neuromodulation, effect intensities (intensity spatial peak temoral average ISPTA.3, intensity spatial peak peak average ISPPA.3), mechanical index (MI) and positive/negative peak pressures typically exceed the FDA limits. In most studies peak pressures lie below 10 MPa with FUS peaks clearly lower than TPS peaks.^[^ ^44^ ^]^ ISPTA.3 values typically reach 1000 mW cm^−2^ with FUS and are clearly lower with TPS (100 mW cm^−2^). A special feature of the ultrashort TPS pulses (about 3 µs) is that they cannot produce thermal bioeffects and secondary maxima via standing waves. The TPS technique is also approved for clinical application (CE certification for Alzheimer's disease) and clinical research (FDA IDE certification). Current data from healthy subjects and patients indicate that ultrasound neuromodulation is safe, and none of the studies has ever described a serious adverse event (SAE). For the system most widely used clinically (TPS), more than 15 000 treatment sessions (typically 1000–6000 TPS pulses per session) have been performed without any serious adverse event (SAE). TPS animal studies indicate, that sonication with 150‐fold energy levels compared to the maximum human dose allowed, do not result in any brain damage.^[^ ^6^ ^]^ Of course, for patient applications it is important to exclude any risks for bleeding and determine the individual patient's clinical state by thorough examinations and high‐resolution MRI images immediately before the treatment. Any pathologies with increased risk for bleeding (e.g., small cavernomas) or patients with blood clotting disorders need to be detected. Mild to moderate adverse events previously reported with ultrasound neuromodulation are similar to adverse events reported for electromagnetic brain stimulation (compare Machii et al.^[^ ^45^ ^]^). In the largest patient observation currently published (*n* = 101, TPS technique, Radjenovic et al.^[^ ^44^ ^]^), intra‐treatment adverse events were reported by about 3% of the patients, post‐treatment events were noted by about 13%. Over all human ultrasound neuromodulation studies ever published, the following mild to moderate adverse events have been reported: localized pain at head or neck, general headache, painless pressure sensations at the stimulation site, muscle twitches, heating sensations, itchiness, anxiety, uncomfortable feelings, mood deterioration, difficulty paying attention, confusion, tenseness, disorientation, noise sensitivity, tingling, nausea, sleepiness, tiredness, dizziness, unsteady gait, tremor worsening, and sweating.^[^ ^4^, ^44^, ^46^, ^47^ ^]^ It is important to realize that adverse events are also reported when sham (placebo) stimulations are applied.^[^ ^47^ ^]^

## 6 Perspectives for Ultrasound Neuromodulation as a New Brain Therapy

Clinical ultrasound neuromodulation with state‐of‐the‐art systems is a new but promising brain therapy. Patient data with the navigated focused state‐of‐the‐art technologies are however limited. Since the first patient series published in 2019 (35 Alzheimer's patients, Beisteiner et al.^[^ ^6^ ^]^), 7 additional studies with a total of 87 verum stimulation patients appeared concerning Alzheimer's, Epilepsy, Disorders of Consciousness and Depression. Considering all 3 technical approaches for ultrasound neuromodulation, a total of 16 clinical studies exists (Table 1).

These data open new therapeutic perspectives since most of the patients studied have already been receiving state‐of‐the art treatments, and clinical improvements are therefore add‐on effects. Currently, clinical research is rapidly increasing. A search in ClinicalTrials.gov indicates at least 17 patient trials are running or intended to be performed. They concern a large spectrum of diseases: temporal lobe epilepsy, depression, treatment‐resistant schizophrenia, opioid‐use disorder, posttraumatic stress disorder, anxiety disorders, obsessive‐compulsive disorder, brain tumor, essential tremor, acute and chronic pain, headache, mild cognitive impairment, dementia, and Parkinson's disease.

The major problem with the current clinical data concerns a lack of sham‐controlled trials. Only 3 studies included a sham control,^[^ ^33^, ^37^, ^41^ ^]^ with 2 of them reporting a significant clinical benefit for verum over sham.^[^ ^33^, ^37^ ^]^ Although there is clear independent evidence from neurophysiological techniques that ultrasound generates local neuromodulation in patients (see above) and healthy individuals (compare Sarica et al.^[^ ^46^ ^]^) and recently the first study showing true more than sham fMRI effects in humans appeared,^[^ ^48^ ^]^ it is yet unknown to what extent changed neuronal activity translates into clinical effects. Literature shows that placebo effects in clinical brain stimulation studies may be very large and typically high patient numbers (>100) are required to achieve a significant verum/placebo difference.^[^ ^49^, ^50^ ^]^ An important reason is that documentation of clinical effects depends on clinical evaluations and rating scales, which often lack sensitivity for small differences and include considerable rater dependency. Therefore, it is of utmost importance to include sham conditions and independent neurophysiological measures (MRI, electrophysiology) in clinical studies. Neurophysiological measures provide a high sensitivity and specificity for detecting clinically relevant true more than sham modulations, and they are less rater dependent. Fortunately, many of the registered clinical trials now include sham stimulation.

Another major problem are the low sample sizes in presently published studies. The largest clinical study included 35 verum patients (Table 1). Due to small effect sizes, inter‐individual patient variability and large placebo effects, sample sizes for clinical ultrasound neuromodulation studies need to be considerably increased. In clinical trials, the problem of inter‐individual variability is particularly significant since pathologies may result in gross and highly variable morphological and functional brain changes. This is also a major issue for individual treatment planning. It is important to realize, that the new focused and navigated stimulation techniques include the potential for a personalized precision medicine. Their exceptionally precise targeting options require rigorous definition of individual clinical deficits and functional network changes in often multi‐morbid patients with multiple diagnoses. For secure and effective clinical application neuronal network pathologies with pathological hyper‐ and hypoactivations of network nodes must be defined. This requires dedicated clinical and clinical neuroscientific expertise. If this is given, application data from TPS treatments show that a clear majority of patients improves in clinical scales (see above, placebo effects included) and judge ultrasound add‐on therapy as very helpful. However, clinical recommendations for ultrasound neuromodulation yet need to be published by specialized physicians from the field.

Concerning further methodological developments, there are several promising perspectives. Initial evidence shows that a differential neuromodulation, namely activation and inhibition might become possible.^[^ ^51^ ^]^ With FUS technologies, many parameters may be changed including fundamental frequency, duty cycle, energy settings, single pulse durations, pulsing structures with primary and secondary pulsing levels, and total sonication times. Target specificity is important and minor differences in targeting and delivery may also produce different results.^[^ ^29^ ^]^ The extensive variables offer a lot of research options but also a lot of interdependencies and difficulties for effect interpretations. With TPS, there are only two parameters, which may be changed: single pulse energy and single pulse frequency. This reduces research options but makes controlled investigations more straightforward. In any case, future investigations on neuromodulatory effects in healthy and diseased participants need to include independent monitoring of neuronal activity like functional MRI or EEG or magnetoencephalography (MEG) measures. Another exciting option for further developments concerns the possibility to combine the strengths of electromagnetic and ultrasound neuromodulation techniques. Closed loop electromagnetic approaches allow cortical neuromodulation of larger brain areas, based on immediate functional feedback.^[^ ^20^ ^]^ Based on the animal data described above, closed loop stimulation may be advanced by the high focality and depth range of FUS/TPS to target focal pathological oscillations in 3D. From a longer perspective, current research in freely moving ultrasound systems^[^ ^52^ ^]^ and in sonogenetics^[^ ^53^ ^]^ may eventually provide further milestones for ultrasound neuromodulation as a new brain therapy.

## Conflict of Interest

R.B. received research and laboratory support from Medical University (SO10300020), FWF KLIF455, STORZ Medical AG, and Herzfelder Stiftung. M.H. is an inventor of a patent held by NIH for the H‐coil for magnetic stimulation; in relation to the patent, he has received license fee payments from the NIH (from Brainsway). He is on the Medical Advisory Board of Brainsway (unpaid position). A.M.L. is a consultant to Medtronic, Boston Scientific, Abbott, Insightec, and Functional Neuromodulation.
